# Automatic Supported Liquid Extraction (SLE) Coupled with HILIC-MS/MS: An Application to Method Development and Validation of Erlotinib in Human Plasma

**DOI:** 10.3390/pharmaceutics2020105

**Published:** 2010-04-01

**Authors:** Jiongwei Pan, Xiangyu Jiang, Yu-Luan Chen

**Affiliations:** 1 Charles River Laboratories, 334 South Street, Shrewsbury, MA 01545, USA; 2 Covance Laboratories, 3301 Kinsman Boulevard, Madison, WI 53704, USA; E-Mail: xiangyu.jiang@covance.com (X.J.); 3 Sepracor Inc., 84 Waterford Drive, Marlborough, MA 01752, USA; E-Mail: yu-luan.chen@sepracor.com (Y.-L.C.)

**Keywords:** supported liquid extraction (SLE), erlotinib, HILIC-MS/MS, human plasma

## Abstract

A novel bioanalytical method was developed and validated for the quantitative determination of erlotinib in human plasma by using the supported liquid extraction (SLE) sample cleanup coupled with hydrophilic interaction liquid chromatography and tandem mass spectrometric detection (HILIC-MS/MS). The SLE extract could be directly injected into the HILIC-MS/MS system for analysis without the solvent evaporation and reconstitution steps. Therefore, the method is simple and rapid. In the present method, erlotinib-*d_6_* was used as the internal standard. The SLE extraction recovery was 101.3%. The validated linear curve range was 2 to 2,000 ng/mL based on a sample volume of 0.100-mL, with a linear correlation coefficient of > 0.999. The validation results demonstrated that the present method gave a satisfactory precision and accuracy: intra-day CV < 5.9% (<8.4% for the lower limit of quantitation, LLOQ) with n = 6 and the accuracy of 98.0–106.0%; inter-day CV < 3.2% (<1.5% for LLOQ) with n = 18 and the accuracy of 100.0–103.2%. A dilution factor of 10 with blank plasma was validated for partial volume analysis. The stability tests indicated that the erlotinib in human plasma is stable for three freeze-thaw cycles (100.0–104.5% of the nominal values), or 24-h ambient storage (100.0–104.8% of the nominal values), or 227-day frozen storage at both -20 °C (91.5–94.5% of the nominal values) and -70 °C (93.3–93.8% of the nominal values). The results also showed no significant matrix effect (<6.3%) even with direct injection of organic extract into the LC-MS/MS system. The validated method has been successfully applied to support a clinical study.

## 1. Introduction

LC-MS/MS has been routinely used to determine drug concentrations in biological matrices to support preclinical and clinical studies [1,2]. An analytical method must be developed and validated to ensure the reliable analysis of clinical specimens. Sample preparation affects multiple aspects of an assay method even with the highly specific tandem mass spectrometric detection. Protein precipitation (PPT), liquid-liquid extraction (LLE), and solid phase extraction (SPE) are among the most commonly used sample cleanup techniques for biological sample analysis. In general, LLE provides cleaner extracts than other approaches. LLE, however, has been typically performed manually in an individual test tube with a relatively large volume of organic solvent. The introduction of a 96-well format with a robotic liquid handling system significantly improved the throughput of LLE [3,4,5,6,7,8,9,10,11,12,13,14,15]. However, a 96-well plate allows only a very limited volume of extraction solvent for each well and relatively gentle vortex-mixing that may not be able to extract the analytes with high efficiency. Moreover, it is fairly easy to cause cross-well contamination. Supported liquid extraction (SLE) is a newly developed sample cleanup technology that is particularly suitable for the 96-well format operation. The SLE cartridge is packed with a modified form of diatomaceous earth. When biological samples are applied to the SLE cartridges, the aqueous portion is deposited as a thin film on the hydrophilic surface. An extraction solvent is then applied to elute analytes. Similar to the traditional LLE, SLE provides very clean extracts with a high recovery. Several SLE bioanalytical LC-MS/MS or GC-MS assays have been developed and validated using SLE packed with diatomaceous earth material [16,17,18,19,20].

In addition to sample preparation, another focus of bioanalytical method development is chromatographic separation. Hydrophilic interaction liquid chromatography (HILIC) has been increasingly gaining in popularity mainly because it provides good retention for polar analytes. Meanwhile, HILIC columns often allow direct injection of organic extract into the LC-MS/MS system so that the dry-down and reconstitution steps could be eliminated without the compromise of chromatographic peak shape. The above advantages have been demonstrated by several examples [21,22,23,24,25]. In this manuscript, we delineate an application case of the combination of the automatic 96-well format SLE sample extraction with direct injection of the organic extract into a HILIC-MS/MS, for the simple and fast measurement of erlotinib in human plasma. The features of this novel approach are demonstrated by the assay development and validation of erlotinib in human plasma. The matrix effect, extraction efficiency and recovery of using SLE extraction and HILIC with MS/MS detection are compared with other routine sample preparation approaches. 

## 2. Experimental Section

### 2.1. Chemicals and reagents

Acetonitrile (ACN) and formic acid (96%) of HPLC-grade were purchased from Sigma-Aldrich (Milwaukee, WI). Concentrated ammonium hydroxide and methyl-*tert* butyl ether (MTBE) both were HPLC-grade and purchased from Fisher Scientific (St. Louis, MO, USA). The reference standard erlotinib hydrochloride was purchased from Toronto Research Chemicals Inc. (Toronto, ON, Canada). The deuterated internal standard (IS) erlotinib-*d_6_* hydrochloride was synthesized at Covance Laboratories (Madison, WI) and the isotope purity of >99.9% was confirmed by ESI-MS and was sufficient for the purpose of intended use. Normal human plasma with potassium EDTA (K_2_EDTA) anti-coagulant was purchased from Biochemed (Winchester, VA, USA).

### 2.2. Instrumentation

The 96-well format supported liquid extraction plate (Isolute^®^ SLE+, 200-µL capacity) was purchased from Biotage (Charlottesville, VA, USA). Plasma sample aliquoting was using a Microman pipette. The addition and transfer of reagent, solvent, and mixed samples were performed by a Tomtec Quadra 96-320 liquid handling system. The HPLC system utilized Shimadzu (Kyoto, Japan) LC-10ADVP pumps, and a Shimadzu SIL-HTc system controller and autosampler. An API 3000 triple quadrupole mass spectrometer operated with the *Analyst*™ software (version 1.4.1) obtained from Applied Biosystems/MDS Sciex (Concord, ON, Canada) was used for MS/MS detection. The same software was also used for data acquisition and result calculation. The analytical column used was a 50 × 3 mm Unison UK-Silica, 3-µm particle size, from Imtakt (Tokyo, Japan). The pre-filter consisted of a 5-µm inlet filter and a 5-µm outlet filter from Upchurch Scientific (Oak Harbor, WA).

### 2.3. Preparation of standard solutions, calibration standards, and quality control (QC) samples

Standards and QC samples were prepared from two separate stock solutions. Stock solutions of erlotinib were prepared by dissolving accurately weighed 1.00 mg erlotinib (free-form) in 10.0 mL of 1:1 ACN-H_2_O. The weighing precision between two stock solutions was checked by LC-MS/MS to ensure stock solutions agreed within 5% for the method validation use. The IS stock solution was prepared at 100 µg/mL in 1:1 ACN-H_2_O. The IS working solution was diluted with the same solvent from the IS stock solution to 1.00 µg/mL. Stock solutions, IS stock solution, and working solutions were stored in polypropylene vials at approximately 4 °C. The erlotinib stock solutions were diluted in pooled negative plasma on each day of analysis to prepare calibration standards. The calibration standards were at concentrations of 2, 4, 12, 50, 200, 800, 1,800, and 2,000 ng/mL. The lower limit of quantitation (LLOQ) and the upper limit of quantitation (ULOQ) of this method were 2 ng/mL and 2,000 ng/mL, respectively. The QC sample pools were prepared independently by spiking standard solution in pooled human plasma at five concentration levels, *i.e.*, 2 (LLOQ), 6 (low), 160 (mid), 1,600 (high), and 5,000 (dilution) ng/mL. These QC samples were aliquoted into pre-labeled polypropylene vials (~0.5 mL per vial) and then stored in a freezer maintained at approximately -70 °C. A small batch of low-, mid-, and high-QC samples were stored at approximately -20 °C for testing sample storage stability under this temperature condition.

### 2.4. Sample extraction

**Figure 1:**
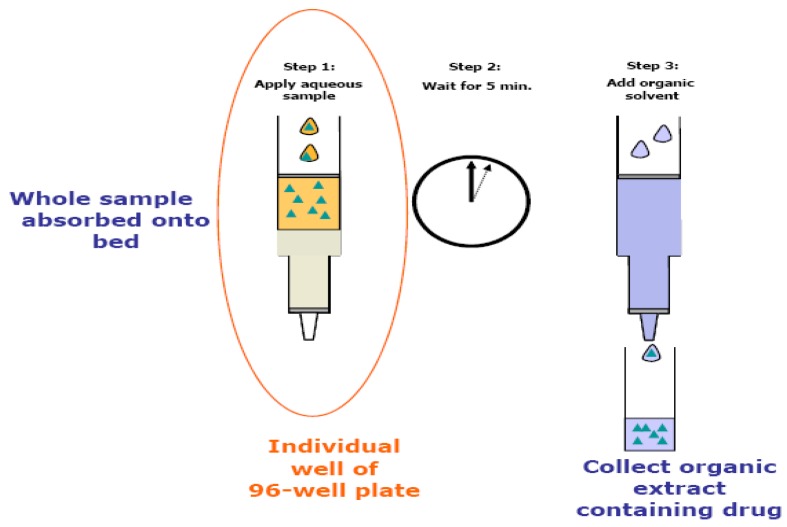
The supported liquid extraction process using the supported liquid extraction (SLE+) cartridge (*adopted from Biotage website www.biotage.com with permission*).

The SLE extraction process is illustrated in Figure 1. An aliquot of 100-µL human plasma sample was mixed with 10 µL of the IS working solution (1.00 µg/mL) in a deep-well 96-well plate. An aliquot of 100 µL 10% ammonium hydroxide was added to each well and vortex-mixed well. A 200-µL aliquot of each mixed sample was applied to a 96-well SLE+ plate. A gentle positive pressure was applied onto each well for a couple of seconds to initiate the absorption process. The samples were allowed to interact with SPE sorbent for about 5 min so the aqueous portion and other reagent components were completely absorbed into the packing material. Then, 0.80 mL of MTBE was added to each well to elute analytes into a clean 96-well collection plate by gravity for a couple of minutes. Finally, a positive pressure was applied to ensure completion of the elution. A 10-µL aliquot of each eluent was injected directly into the HILIC-MS/MS for analysis.

### 2.5. LC-MS/MS conditions

Separation of the analyte from potential interference in the matrix was achieved on a 50 × 3 mm Unison UK-Silica column at ambient temperature. An isocratic mobile phase consisting of 0.1% formic acid in 85:15 (v:v) ACN-water was used at a flow-rate of 1.0 mL/min. A 1:1 ACN-water solution was used as the injector wash solvent. MS/MS detection was performed using a Sciex API 3000 triple quadrupole mass spectrometer with a Turbo Ionspray^®^ ionization source operated in the positive ion mode. The mass spectrometry parameters such as analyte fragmentation pattern and collision energy, *etc*. were optimized by infusing the analyte and the IS solutions. The LC-MS interface conditions such as gas flows, source temperature, *etc.* were optimized *via* tee-mixing the analyte standard solution with the mobile phase at a flow-rate of 1.00 mL/min. All optimized parameters for this method are presented in Table 1.

**Table 1:** Tandem mass spectrometric parameters for the LC-MS/MS assay.

Source temperature (°C)	500
Dwell time per transition (ms)	150
Nebulizer gas (psi)	12
Auxiliary gas (L/min)	8
Curtain gas setting	10
Collision gas setting	12
IonSpray voltage (V)	2,000
Declustering potential (V)	35
Focusing potential (V)	210
Collision energy (eV)	45
Collision Cell Exit Potential (V)	25
Resolution for Q1 and Q3	Unit
Mode of analysis	Positive
Ion transition for erlotinib, m/z	394→278
Ion transition for IS, erlotinib-*d_6_*, m/z	400→278

### 2.6. Quantitation and assay validation

The peak areas of the analyte and the IS were integrated using *Analyst^®^* software version 1.4.1. For each analytical run, a calibration curve was derived from analyte/IS peak area ratio against the analyte concentration using a weighted (1/x^2^) linear least-squares regression. The regression equation was then used to calculate the concentration of human plasma samples. During the method validation, intra-day and inter-day precision (CV%) and accuracy (RE% or percent of theoretical value) were calculated. The presented method was validated following the FDA guidance for bioanalytical method validation [26] for intra-day and inter-day precision and accuracy, linearity, selectivity, sensitivity, dilution integrity, and short-term and long-term sample stability. 

## 3. Results and Discussion

### 3.1. Mass spectrometry

**Figure 2:**
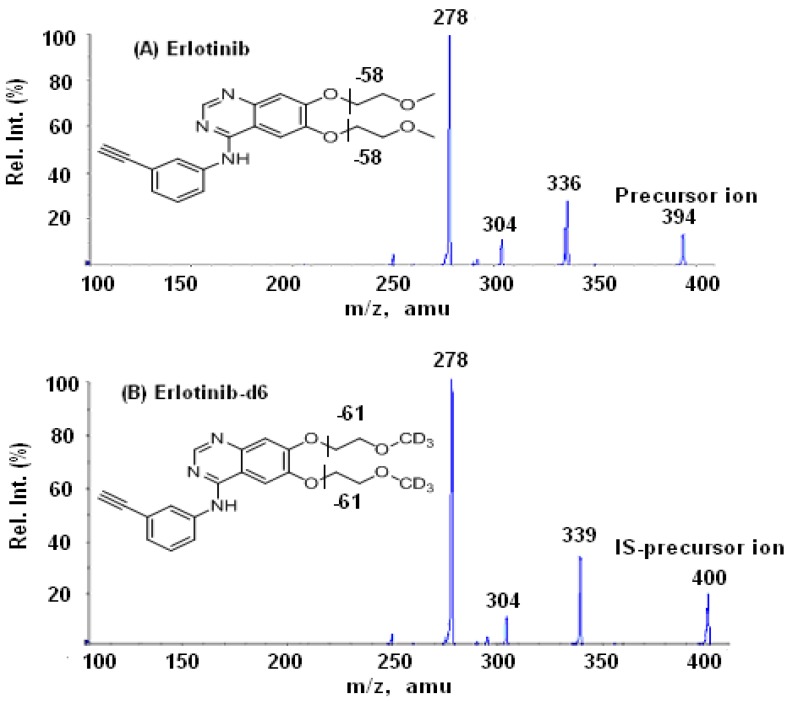
Mass spectra of erlotinib (**A**) and erlotinib-*d_6_* (**B**) and their fragmentation pathways.

The protonated parent and product ions of erlotinib and the IS are shown in Figure 2A and Figure 2B. The most abundant product ions obtained from both the analyte and IS were *m/z* 278. Other major product ions included 336 from erlotinib and *m/z* 339 from the IS, erlotinib-*d_6_*. This implied that the precursor ions could be broken down by loss of one or two –CH_2_-CH_2_-O-CH_3_ (or –CH_2_-CH_2_-O-CD_3_) groups from two branch chains, where erlotinib-*d_6_* was labeled with deuterium. Given that the *m/z* 278 ion was a predominant product ion for both the analyte and IS, the transitions 394→278 and 400→278 were selected for MRM monitoring of the analyte and the IS, respectively.

### 3.2. Chromatography

**Figure 3:**
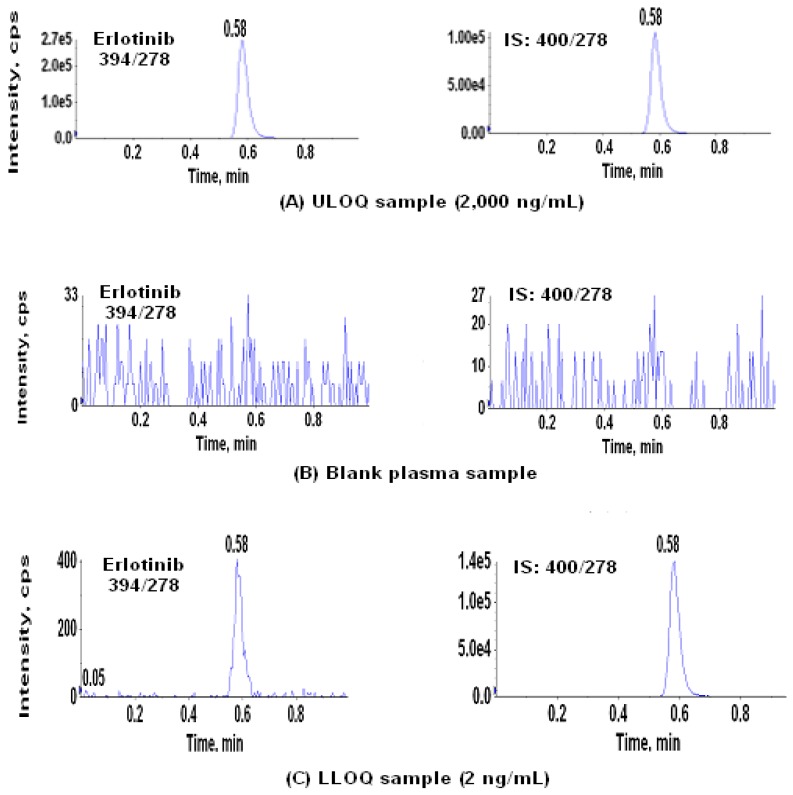
Typical chromatograms obtained from SLE extracted samples: (A) ULOQ sample (2,000 ng/mL), (B) blank plasma sample, and (C) LLOQ sample (2 ng/mL). (The matrix blank sample was injected immediately after an ULOQ, therefore also an indicative of no carryover. Left panel – Erlotinib; Right panel – Internal standard, IS).

Chromatographic separation was carried out on a 50 × 3 mm silica column (Unison UK-Silica) with a mobile phase of 85:15:0.1 (v:v:v) ACN-water-formic acid at a flow-rate of 1.0 mL/min. The use of such a high organic content mobile phase resulted in a low backpressure at ~40 bars. The HILIC system allowed direct injection of the eluate resulting from the SLE sample preparation procedure. Because it has been proven that erlotinib is insoluble in pure acetonitrile, for the injector wash and stock solution preparation, a mixture of 1:1 ACN-water was used for both the prevention of potential carryover from the needle and dissolving erlotinib and the IS material. No carryover from the injection needle was observed during the method validation. In addition, to minimize the evaporation of the MTBE in the extracts, the autosampler cooling system was set at 4 °C. Retention times for both erlotinib and its IS were approximately 0.58 min. A cycle time of about 1 min per sample is several times faster than the published methods [27,28,29]. The capacity factor *k’* for the analyte peak was about 1.5 (the column dead volume is about 0.23 mL and *k’* = (0.58-0.23)/0.23 = 1.5), indicating that the analyte has a sufficient on-column retention for analysis. The representative chromatograms of ULOQ, matrix blank, and LLOQ samples are shown in Figure 3. The clean chromatogram of the matrix blank obtained from the injection of a matrix blank extract immediately after an ULOQ sample demonstrated that this method had neither injector carryover nor analytical column carryover.

### 3.3. Comparison of SLE with protein precipitation, and liquid-liquid extraction

The protein precipitation (PPT) approach was evaluated first due to its simplicity. An aliquot of 100 µL of plasma sample was vortex-mixed with 400 µL of ACN-methanol-formic acid (50:50:0.15, v:v:v) and then centrifuged for 5 min. A 50.0-µL aliquot of supernatant was diluted with 400 µL ACN prior to injection. A noisy chromatographic baseline was noticed in the chromatogram resulted from the PPT-pretreated sample. However, SLE-extracted sample had clean baseline and a nice analyte peak, indicating that SLE is a cleaner procedure than PPT. The PPT procedure yielded an overall extraction recovery of 73.3%, which was about 28% lower than that obtained from the described SLE procedure (see Table 2).

The 96-well format SLE was also compared with a traditional LLE in a 96-well format plate. Both SLE and LLE used the same amount of organic solvent, *i.e.,* 0.8-mL MTBE, for extraction. In comparison with the traditional LLE, the overall extraction efficiency by SLE was about 18% higher than the classic LLE (see Table 2). The SLE extraction recovery was nearly 100%, indicating the SLE is an ideal extraction approach for erlotinib from human plasma. In addition, the SLE also showed almost no suppression for analyte ionization for the mass spectrometric detection.

**Table 2:** Extraction recovery of erlotinib from plasma: SLE, LLE and PPT.

Extraction method	PPT*^a^*			LLE*^b^*			SLE*^c^*		
Plasma conc. (ng/mL)	6	160	1,600	6	160	1,600	6	160	1,600
Extraction recovery (%) (n = 6)	69	73	78	89	83	79	104	97	103
Overall mean recovery (%)	73.3			83.6			101.3		

a - PPT: protein precipitation with 0.4-mL acetonitrile-methanol-formic acid (50:50:0.15);

b - LLE: liquid-liquid extraction with 0.8-mL MTBE;

c - SLE: supported liquid extraction with 0.8-mL MTBE for elution.

### 3.4. Linear curve range and assay sensitivity (LLOQ)

The linearity of the calibration curve was evaluated from three individual batches on three different days. The linear dynamic range for erlotinib was from 2.00 to 2,000 ng/mL based on a 100-µL plasma. The coefficient of determination (r^2^) of the calibration curve was between 0.9973 and 0.9995 and the mean back-calculated concentrations of standards ranged from 93.0% to 105.3% of the theoretical values (Table 3). Eighteen replicates (six replicates in each of three batches) of the lower limit of quantitation (LLOQ) samples were used to evaluate the inter-day precision and accuracy at the low end of the assay range in three separate runs. The inter-day CV% was 1.5% (n = 18) and the accuracy, expressed as percent theoretical, was 101.5% (Table 4). The representative LC-MS/MS chromatogram of an LLOQ sample is shown in Figure 3C.

**Table 3:** Back-calculated erlotinib calibration standards in human plasma.

Calibrator (ng/mL)		Back-calculated conc. (mean ± SD) (ng/mL)	Precision (CV%, n = 3)	Accuracy (% nominal)
2		2.06 ± 0.06	2.7	103.0
4		3.72 ± 0.24	6.3	93.0
10		12.5 ± 0.06	0.5	104.2
50		50.1± 0.7	1.4	100.2
200		197 ± 3.2	1.6	98.5
800		842 ± 11.7	1.4	105.3
1,800		1740 ± 5.6	0.3	96.7
2,000		2070 ±76	3.8	103.5

**Table 4:** Intra-day and inter-day precision and accuracy of quality control samples including LLOQ and the dilution QC* samples.

QC sample (ng/mL)	Intra-day					Inter-day			
	LLOQ 2.00	LQC 6.00	MQC 160	HQC 1,600	DiQC 5,000	LLOQ 2.00	LQC 6.00	MQC 160	HQC 1,600
N	6	6	6	6	6	18	18	18	17
Mean	1.96	6.04	159	1,630	2.03	6.19	6.19	160	1,640
CV (%)	8.4	5.9	3.9	3.6	2.8	1.5	3.2	4.0	2.5
Accuracy (%)	98.0	100.7	99.4	101.9	106.0	101.5	103.2	100.0	102.5

* 10-fold dilution with the control matrix applied to the DiQC (5,000 ng/mL).

### 3.5. Precision, accuracy, and dilution integrity

Six replicates of QC samples for each of three consecutive runs were used to evaluate the intra-day and inter-day precision and accuracy at low-, mid-, and high-QC concentration levels. As shown in Table 4, the intra-day CV (n = 6) was ≤8.4% and the inter-day CV (n = 18, except for HQC n = 17) was ≤2.5%. The mean accuracy of intra-day and inter-day assays was between 99.4–101.9% and 100.0–103.2%, respectively. To validate partial-volume assay, a dilution factor of 10 with blank plasma was processed with the dilution QC sample (5,000 ng/mL). A 10 µL of the dilution QC was mixed with 90 µL of blank matrix in the 96-well sample plate. Results for the dilution QC (DiQC) samples showed a mean accuracy of 106% theoretical. The corresponding CV was 2.8% (n = 6). These results indicate that the present method has satisfactory precision, accuracy and dilution integrity.

### 3.6. Selectivity and matrix effect

Selectivity was evaluated by extracting blank matrix from six different lots and comparing the MS/MS response at the retention times of the analyte to the responses of the LLOQ. No significant peak was observed in any of these 6 lots of blank plasma samples for analyte and IS. The matrix effect on the responses for both erlotinib and its IS were investigated by preparing a mid-QC level with six different individual lots of plasma. The matrix effects were evaluated by comparing the peak area obtained from the post-extraction spiked sample to a pure solution at the same nominal concentration. Matrix effect was calculated as ME (%) = [1 – peak area of post-extraction spiked sample/peak area of pure solution] × 100. With this SLE procedure and chromatographic conditions, the matrix effects for erlotinib and erlotinib-*d_6_* were 3.9% and 6.3%, respectively (individual data not shown). This experiment was also performed using a calibration curve generated from the same set of standards used for the determination of linearity, precision, and accuracy. When quantitated by this curve, the measured results of the matrix effect QC samples in six individual plasma lots were between 97.5% and 110.6% (data not shown). This demonstrates that the matrix lot-to-lot variation is insignificant.

### 3.7. Sample stability

The short-term (freeze-thaw and ambient storage), long-term frozen storage stability at two different temperatures, and extracted samples’ reinjection viability were tested and the results are presented in Table 5. As shown in Table 5, the plasma QC samples were stable for three freeze-thaw cycles (observing 100–104.5% of nominal values), or for a 24-h ambient storage (observing 100–104.8% of nominal values), or for a 227-day frozen storage at both -20 °C (observing 91.9–94.5% of nominal values) and -70 °C (observing 93.3–93.8% of nominal values). Longer-term storage period may be further extended, but has not been confirmed yet at this time. A batch consisting of extracted standards and QC samples were re-injected into the LC/MS/MS system after the 72-h storage in the autosampler condition (4 °C). Both the calibration standard curve and the QC samples results met the pre-set acceptance criteria. The mean accuracy of re-injected QC samples was between 95.6% and 99.2% of their nominal values, indicating that the extracted samples could be re-injected within 72 h when stored under the autosampler condition. The stock solution was stable for 224 days when stored under the refrigerated condition (data not shown).

**Table 5:** Plasma sample short-term, long-term sample stability and extracted samples’ re-injection viability.

Sample Concentration	Mean conc. found (ng/mL)	Precision (CV%, n = 6)	% nominal conc.
Plasma sample ambient storage (24 h)			
Low-QC: 6 ng/mL	6.29	2.0	104.8
High-QC: 1,600 ng/mL	1,600	1.3	100.0
Freeze-thaw cycles (n = 3)			
Low-QC: 6 ng/mL	6.27	3.5	104.5
High-QC: 1,600 ng/mL	1,600	1.7	100.0
Extracted samples at autosampler viability (72 h)			
Low-QC: 6 ng/mL	5.95	2.7	99.2
High-QC: 1,600 ng/mL	1,530	2.2	95.6
Long-term stability in plasma at -70°C for 227 days			
Low-QC: 6 ng/mL	5.67	5.7	94.5
High-QC: 1,600 ng/mL	1,470	4.4	91.9
Long-term stability in plasma at -20°C for 227 days			
Low-QC: 6 ng/mL	5.60	2.6	93.3
High-QC: 1,600 ng/mL	1,501	2.2	93.8

### 3.8. Application

**Figure 4:**
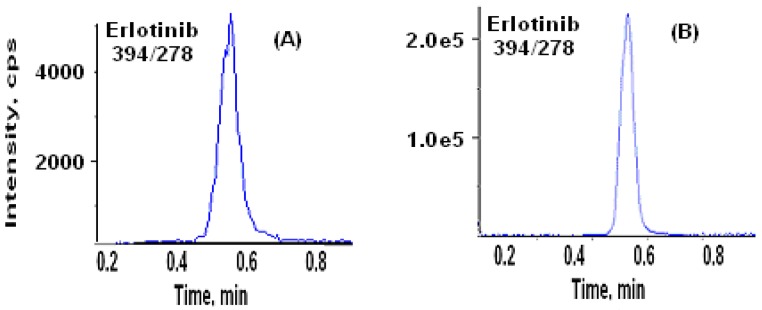
Representative chromatograms of Erlotinib obtained from 24 h (**A**) and 15 min (**B**) post-dose plasma samples (the IS chromatograms are not shown).

The above newly validated method has been applied to support clinical sample analysis. One trough (24 h post-dose) sample and one 15 min post-dose sample from a clinical trial with a single oral dose of 150-mg erlotinib were assayed in six replicates. Then standard addition approach was used to evaluate the assay performance. The chromatograms of these two incurred samples are presented in Figure 4 (the IS chromatograms are not shown). The sample analysis replicates data and standard addition recovery results are presented in Table 6. The satisfactory precision and percent recovery of standard addition samples indicate that this newly developed and validated method provides a reliable quantitation approach for the measurement of erlotinib in human plasma.

**Table 6:** Incurred sample analysis and recovery of standard addition samples.

Plasma Timepoint	Erlotinib Concentration in Human Plasma (ng/mL)			
	24 h post-dose		15 min post-dose	
	Plasma sample	Standard addition: 20.0 ng/mL	Plasma sample	Standard addition: 800 ng/mL
				
Individual measurement	20.1	39.6	779	1590
	19.8	39.8	798	1660
	20.0	38.6	750	1630
	20.1	42.0	824	1650
	20.1	41.6	734	1640
	22.2	42.8	708	1560
Mean	20.4	40.7	766	1622
%CV (n = 6)	3.0	3.4	5.6	2.4
% recovery of standard addition	-	101.5	-	107

## 4. Conclusions

As discussed above, the combination of SLE sample extraction with HILIC-MS/MS provides a simple, fast, sensitive, yet precise and accurate assay method for the quantitative measurement of erlotinib in human plasma samples. The SLE is a cleaner sample preparation procedure with better extraction efficiency than the protein precipitation, and also shows a higher extraction recovery than a traditional liquid-liquid extraction approach with the same volume of the used organic solvent. The use of HILIC column allows the direct introduction of the SLE extract, which further simplify the sample analysis procedure that could eliminate the dry-down and reconstitution steps. In comparison with the published methods [27,28,29], the presented method has reduced the sample preparation time and chromatographic run time significantly. Moreover this novel method has a 50-fold lower LLOQ than the HPLC method [29] and higher extraction recovery (near 100%) than the protein precipitation [28] and the liquid-liquid extraction [27]. No doubt, the current method provides an excellent alternative in support of any erlotinib-related clinical trials, particularly suitable for the studies with large number of PK plasma samples and requiring quick data turnaround. 
